# Real-world exhaust temperature and engine load distributions of on-road heavy-duty diesel vehicles in various vocations

**DOI:** 10.1016/j.dib.2018.04.044

**Published:** 2018-04-18

**Authors:** Kanok Boriboonsomsin, Thomas Durbin, George Scora, Kent Johnson, Daniel Sandez, Alexander Vu, Yu Jiang, Andrew Burnette, Seungju Yoon, John Collins, Zhen Dai, Carl Fulper, Sandeep Kishan, Michael Sabisch, Doug Jackson

**Affiliations:** a University of California at Riverside, United States; b infoWedge, United States; c California Air Resources Board, United States; d US Environmental Protection Agency, United States; e Eastern Research Group, Inc., United States

## Abstract

Real-world vehicle and engine activity data were collected from 90 heavy-duty vehicles in California, United States, most of which have engine model year 2010 or newer and are equipped with selective catalytic reduction (SCR). The 90 vehicles represent 19 different groups defined by a combination of vocational use and geographic region. The data were collected using advanced data loggers that recorded vehicle speed, position (latitude and longitude), and more than 170 engine and aftertreatment parameters (including engine load and exhaust temperature) at the frequency of one Hz. This article presents plots of real-world exhaust temperature and engine load distributions for the 19 vehicle groups. In each plot, both frequency distribution and cumulative frequency distribution are shown. These distributions are generated using the aggregated data from all vehicle samples in each group.

**Specifications Table**

**Table:** 

Subject area	*Engineering*
More specific subject area	*Emissions control from diesel engines*
Type of data	*Graph*
How data was acquired	*The data were collected from 90 heavy-duty vehicles using J1939 Mini Logger*^*TM*^*produced by HEM Data.*
Data format	*Analyzed*
Experimental factors	*The 90 vehicles represent 19 different groups defined by a combination of vocational use and geographic region. Almost all of the vehicles have engine model year 2010 or newer and are equipped with SCR.*
Experimental features	*The data collection effort spanned from November 2014 to September 2016, but was intermittent depending on when the vehicles and data loggers were available. For each vehicle, the data were collected for a minimum period of one month. The collected data include vehicle speed, position (latitude and longitude), and more than 170 engine and aftertreatment parameters at the frequency of one Hz.*
Data source location	*All the vehicles are domiciled and operated mostly in California, United States.*
Data accessibility	*The data are provided in this article.*
Related research article	*Boriboonsomsin, K., Durbin, T., Scora, G., Johnson, K., Sandez, D., Vu, A., Jiang, Y., Burnette, A., Yoon, S., Collins, J., Dai, Z., Fulper, C., Kishan, S., Sabisch, M., and Jackson, D. (2018). “Real-world exhaust temperature profiles of on-road heavy-duty diesel vehicles equipped with selective catalytic reduction.” Science of the Total Environment, accepted on Mar 29, 2018.*

**Value of the data**

• The data allows for a comparison of real-world exhaust temperature and engine load distributions by vocation.
• The data can be compared with other data from different locations and new data collected in future works.
• The exhaust temperature distributions can be used to analyze the potential NOx conversion efficiency of different types of SCR, as done in Ref. [1].
• The data can be used to support the design of exhaust aftertreatment systems for heavy-duty diesel vehicles in specific vocations.

## 1 Data

The data includes plots of real-world exhaust temperature and engine load distributions for the 19 different groups of on-road heavy-duty vehicles in California as defined by a combination of vocational use and geographic region (Fig. 1, Fig. 2, Fig. 3, Fig. 4, Fig. 5, Fig. 6, Fig. 7, Fig. 8, Fig. 9, Fig. 10, Fig. 11, Fig. 12, Fig. 13, Fig. 14, Fig. 15, Fig. 16, Fig. 17, Fig. 18, Fig. 19). In each plot, both frequency distribution and cumulative frequency distribution are shown. These distributions are generated using the aggregated data from all vehicle samples in each group. Note that the exhaust temperature here is referred to the exhaust gas temperature at the inlet of SCR.

**Fig. 1:**
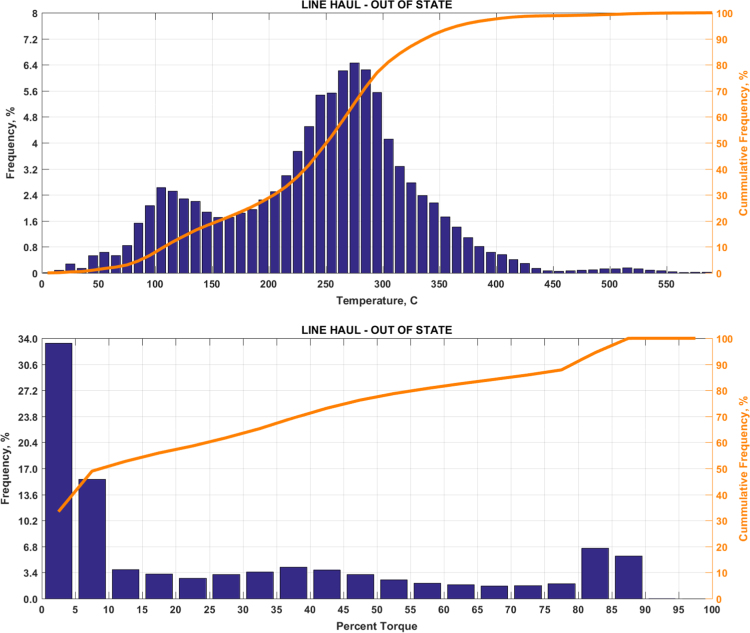
(Top) real-world exhaust temperature distributions and (bottom) engine load distributions of Group 1a (Line haul – out of state).

**Fig. 2:**
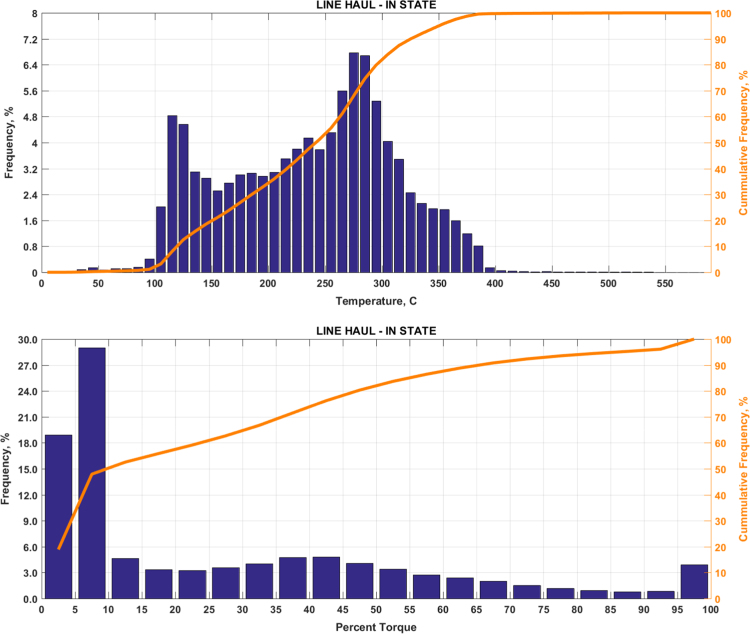
(Top) real-world exhaust temperature distributions and (bottom) engine load distributions of Group 1b (Line haul – in state).

**Fig. 3:**
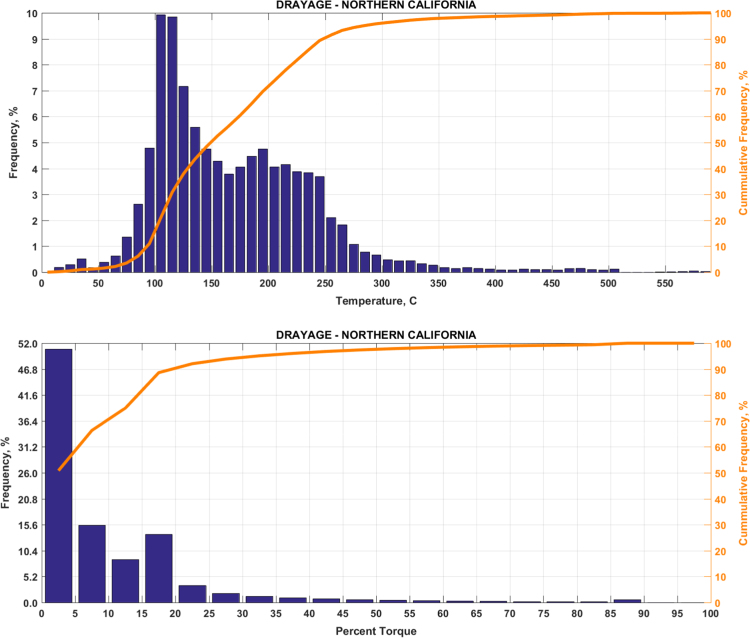
(Top) real-world exhaust temperature distributions and (bottom) engine load distributions of Group 2a (Drayage – Northern California).

**Fig. 4:**
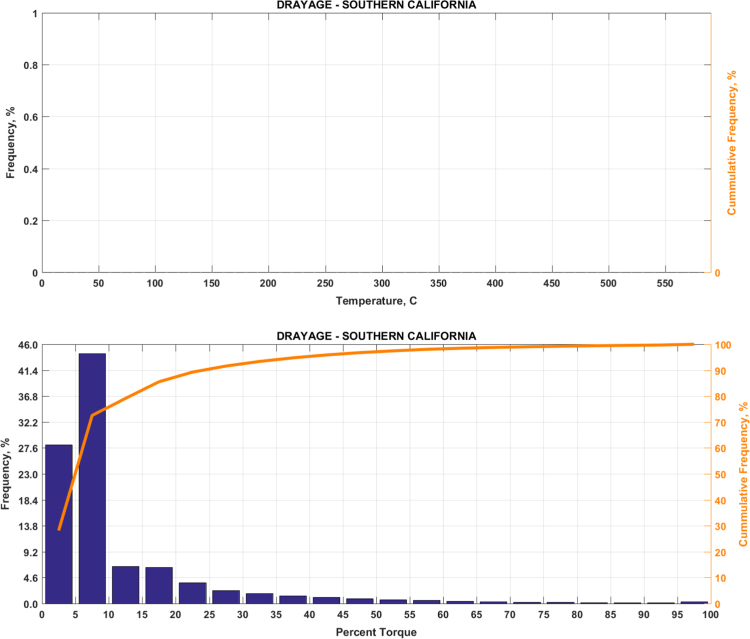
(Top) real-world exhaust temperature distributions and (bottom) engine load distributions of Group 2b (Drayage – Southern California).

**Fig. 5:**
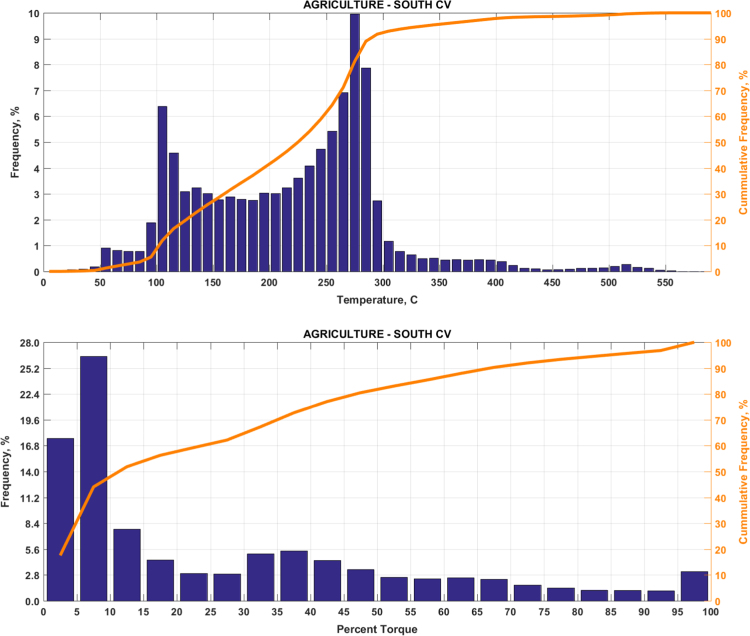
(Top) real-world exhaust temperature distributions and (bottom) engine load distributions of Group 3 (Agricultural).

**Fig. 6:**
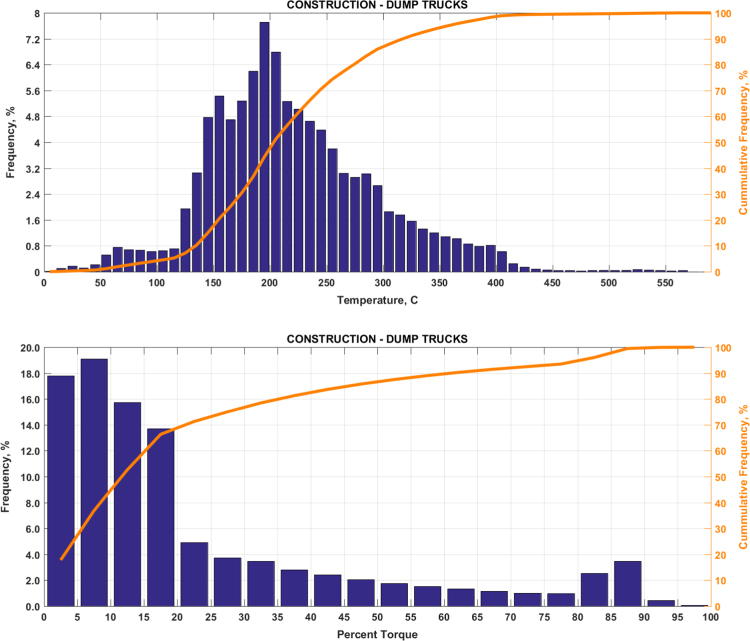
(Top) real-world exhaust temperature distributions and (bottom) engine load distributions of Group 4a (Construction).

**Fig. 7:**
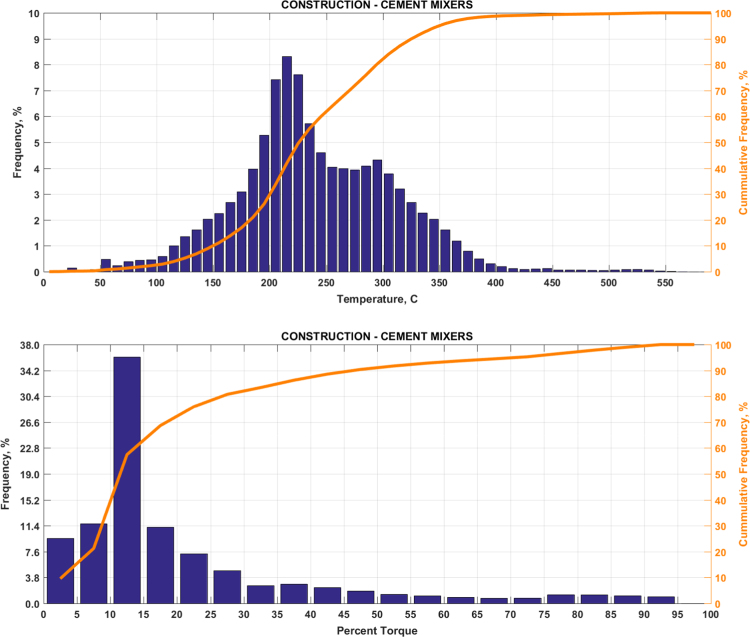
(Top) real-world exhaust temperature distributions and (bottom) engine load distributions of Group 4b (Concrete mixers).

**Fig. 8:**
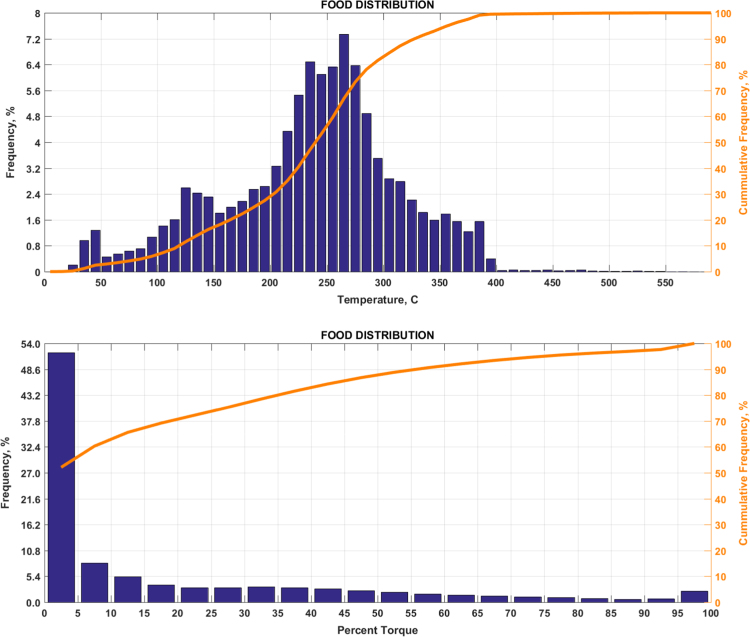
(Top) real-world exhaust temperature distributions and (bottom) engine load distributions of Group 5a (Food distribution).

**Fig. 9:**
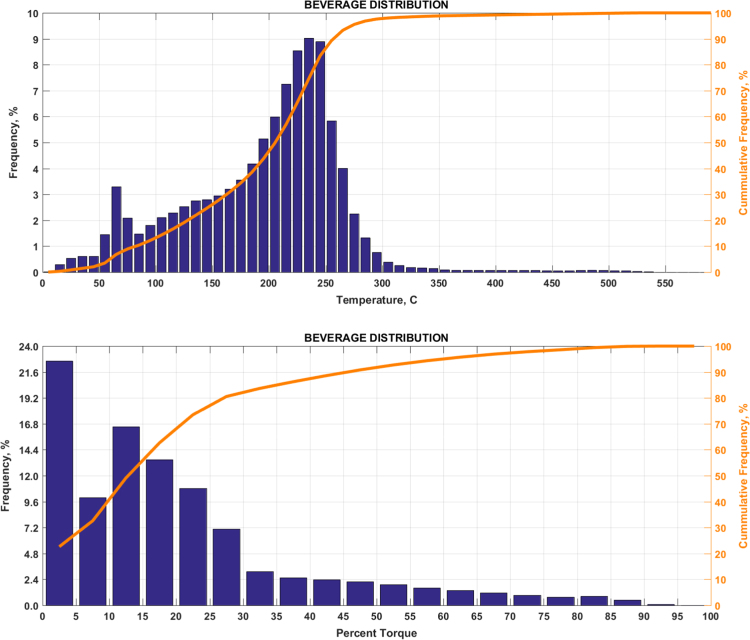
(Top) real-world exhaust temperature distributions and (bottom) engine load distributions of Group 5b (Beverage distribution).

**Fig. 10:**
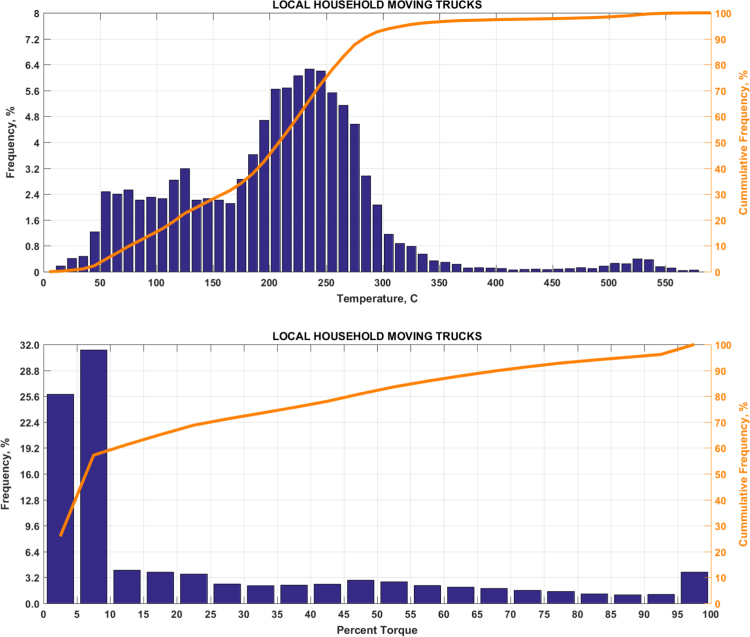
(Top) real-world exhaust temperature distributions and (bottom) engine load distributions of Group 5c (Local moving).

**Fig. 11:**
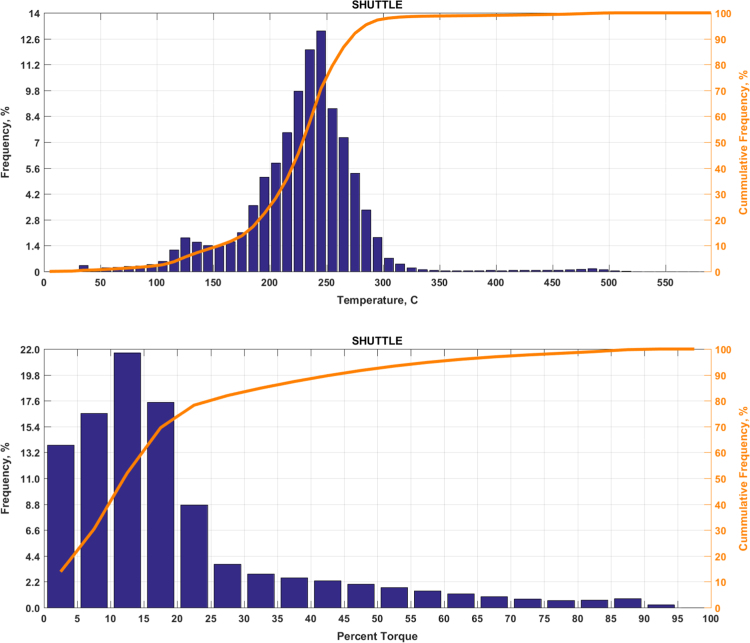
(Top) real-world exhaust temperature distributions and (bottom) engine load distributions of Group 6 (Airport shuttle).

**Fig. 12:**
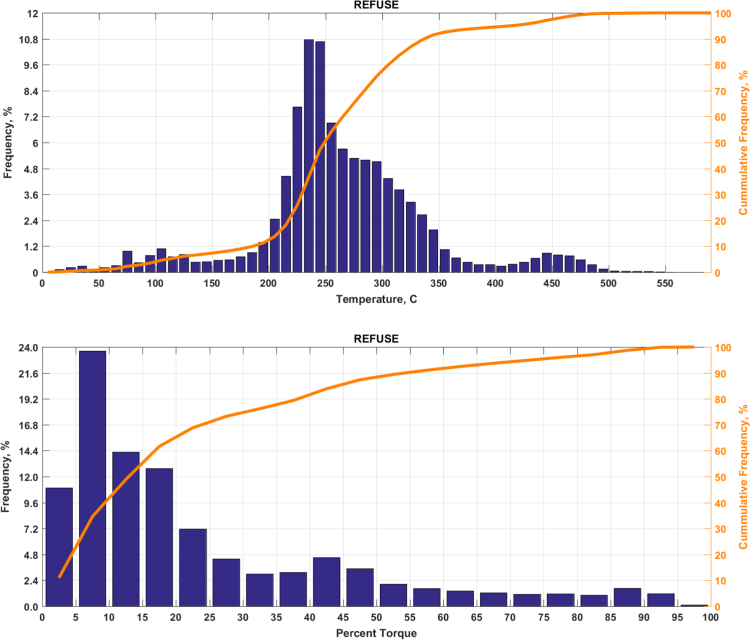
(Top) real-world exhaust temperature distributions and (bottom) engine load distributions of Group 7 (Refuse).

**Fig. 13:**
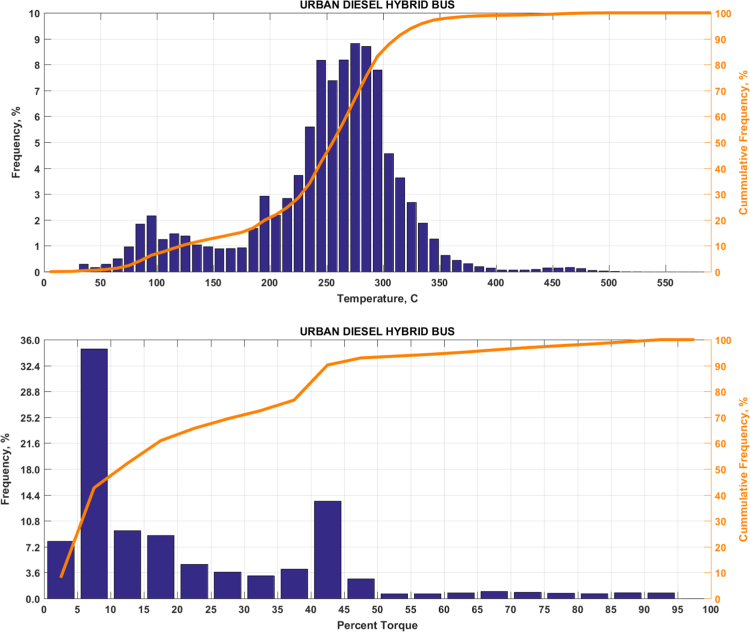
(Top) real-world exhaust temperature distributions and (bottom) engine load distributions of Group 8a (Urban buses).

**Fig. 14:**
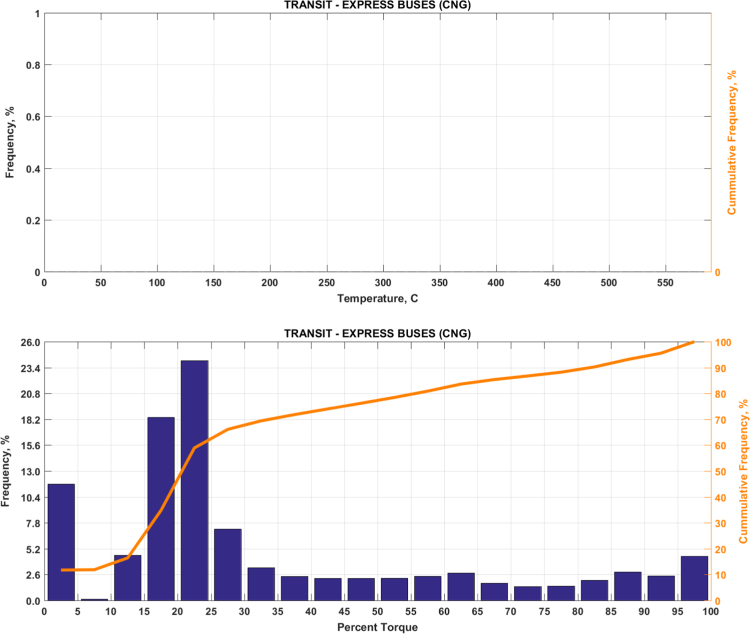
(Top) real-world exhaust temperature distributions and (bottom) engine load distributions of Group 8b (Express buses).

**Fig. 15:**
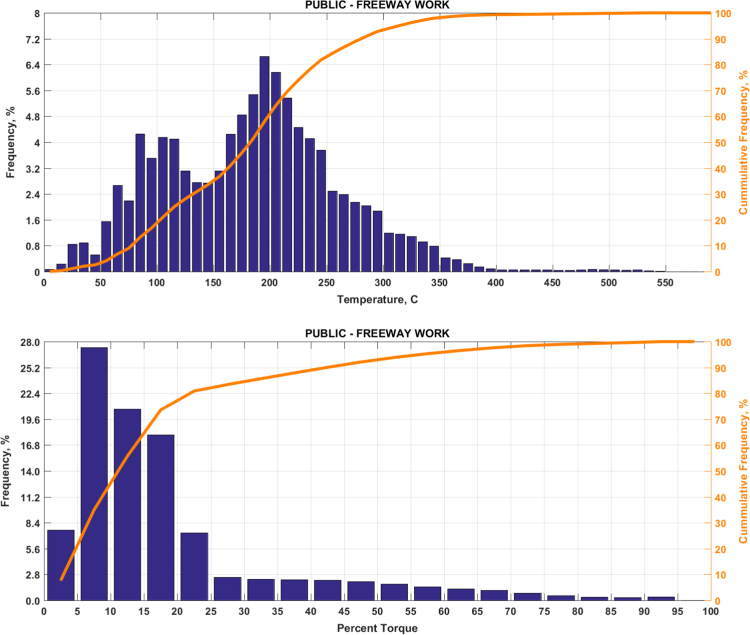
(Top) real-world exhaust temperature distributions and (bottom) engine load distributions of Group 9a (Freeway work).

**Fig. 16:**
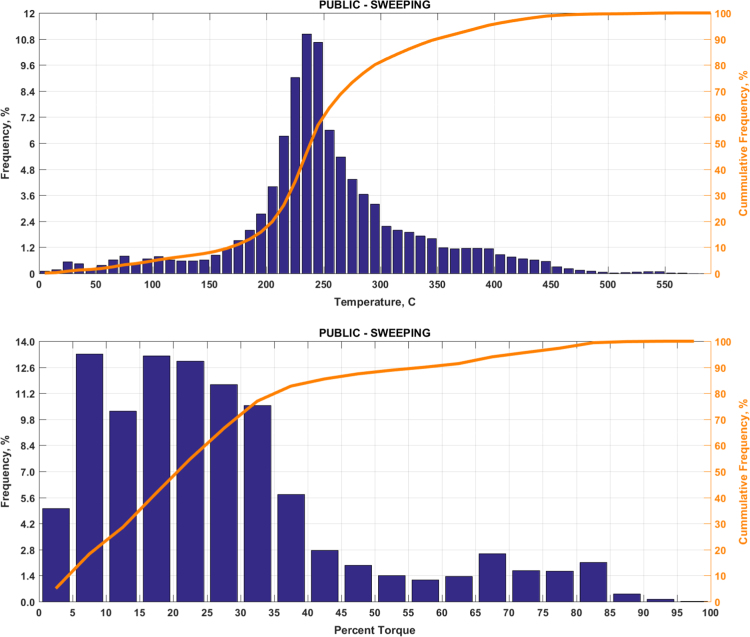
(Top) real-world exhaust temperature distributions and (bottom) engine load distributions of Group 9b (Sweeping).

**Fig. 17:**
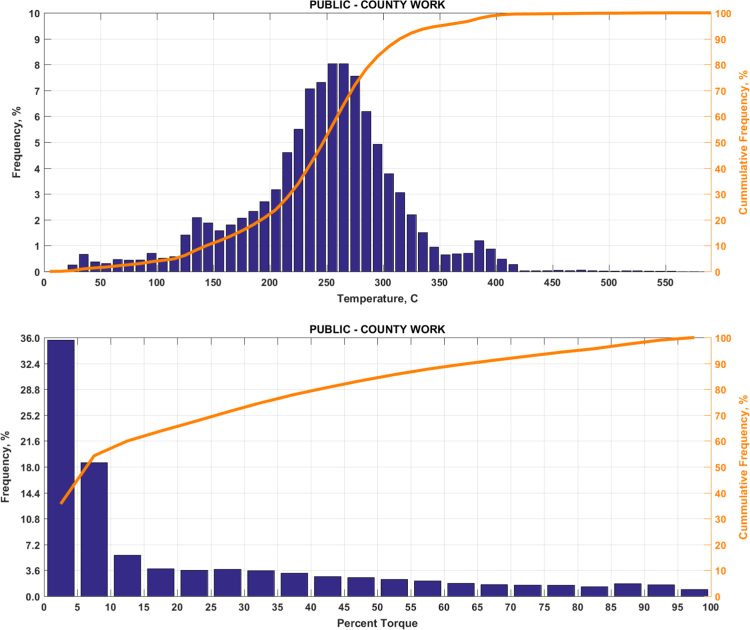
(Top) real-world exhaust temperature distributions and (bottom) engine load distributions of Group 9c (Municipal work).

**Fig. 18:**
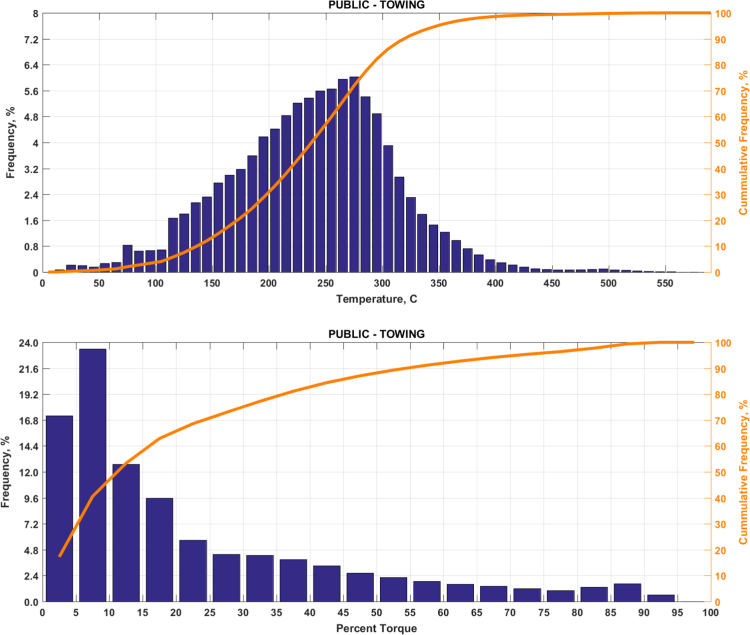
(Top) real-world exhaust temperature distributions and (bottom) engine load distributions of Group 9d (Towing).

**Fig. 19:**
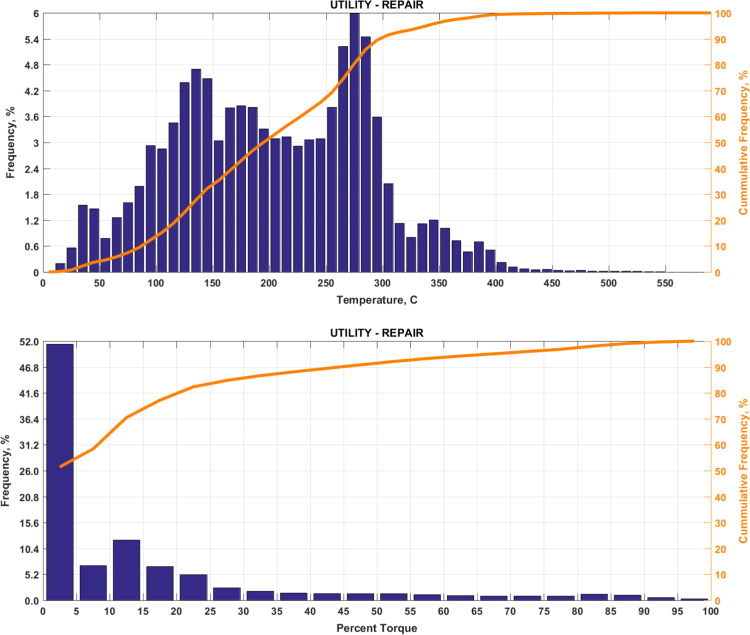
(Top) real-world exhaust temperature distributions and (bottom) engine load distributions of Group 10 (Utility repair).

## 2 Experimental design, materials, and methods

The research team targeted data from 100 vehicles that are domiciled in the state of California, and designed a vehicle sample matrix that balanced between the number of vocations and the number of vehicles in each vocation. The targeted vehicles are from commonly found vocations that, collectively, represent the majority of the NO_x_ emission inventory of heavy-duty diesel vehicles in California [2]. Due to various reasons, such as not being able to recruit vehicles (or a specific number of vehicles) in some groups, lost data loggers, etc., the final dataset includes 90 vehicle samples in 19 groups defined by a combination of vocational use and geographic region as listed in Table 1.

**Table 1:** Information about vehicle samples in each group.

**Vehicle group**					**Engine**				
**ID**	**Name**	**No. of Flt.**	**Fleet location**^a^	**No. of veh.**	**ID**	**Make**	**Model**	**Model year**	**HP**
1a	Line haul - out of state	1	North	3	18	Cummins	ISX15 450	2012	450
					19	Cummins	ISX15 450	2013	450
					20	Cummins	ISX15 450	2014	450
1b	Line haul - in state	1	South	3	114	Detroit Diesel	DD15AT	2015	505
					116	Detroit Diesel	DD13	2015	500
					117	Detroit Diesel	DD13	2015	500
2a	Drayage - Northern California	1	North	1	99	Cummins	ISX15 450	2012	450
2b	Drayage - Southern California	1	South	5	73^b^	MACK	MP8–415C	2012	415
					75^b^	MACK	MP8–415C	2012	415
					76^b^	MACK	MP8–415C	2012	415
					78^b^	MACK	MP8–415C	2012	415
					79^b^	Detroit Diesel	Series 60	2008	n/a
3	Agricultural	1	South	8	85^b^	Paccar	MX	2010/11	n/a
					86^b^	Paccar	MX	2010/11	n/a
					87^b^	Paccar	MX	2012	455
					88	Paccar	MX-13	2014	455
					89^b^	Mercedez-Benz (Detroit Diesel)	OM 460 LA CID 781	2009	450
					90^b^	Mercedez-Benz (Detroit Diesel)	OM 460 LA CID 781	2009	450
					91	Paccar	MX-13	2014	455
					92	Paccar	MX-13	2014	455
4a	Construction	3	Both	6	1	Cummins	ISB6.7 240	n/a	240
					55	Cummins	ISL 300	n/a	300
					56	Cummins	ISL 300	n/a	300
					80	Cummins	ISX15 485	2011	485
					81	Cummins	ISX15 550	2015	550
					82	Cummins	ISX15 550	2015	550
4b	Concrete mixers	2	Both	5	83	Cummins	ISL9 350	n/a	350
					84	Cummins	ISL9 350	n/a	350
					111	Cummins	ISL9 370	2013	370
					112	Cummins	ISL9 370	2013	370
					113	Cummins	ISL9 370	2013	370
5a	Food distribution	1	South	5	50	Detroit Diesel	DD13	2013	500
					51	Detroit Diesel	DD13	2013	500
					52	Detroit Diesel	DD13	2013	500
					53	Detroit Diesel	DD13	2013	500
					54	Detroit Diesel	DD13	2013	500
5b	Beverage distribution	1	South	6	9	Paccar	PX-9	2003	n/a
					10	Cummins	ISX11.9 370	2011	370
					13	Paccar	PX-9	2013	n/a
					14	Paccar	PX-8	2012	n/a
					16	Paccar	PX-9	2013	n/a
					17	Paccar	PX-8	2012	n/a
5c	Local moving	1	South	1	49	Navistar	A410	2013	410
6	Airport shuttle	1	North	5	57	Cummins	ISL	2012	n/a
					58	Cummins	ISL	2012	n/a
					59	Cummins	ISL	2012	n/a
					60	Cummins	ISL	2012	n/a
					61	Cummins	ISL	2012	n/a
7	Refuse	1	North	6	24	Cummins	ISL	2010	380
					25	Cummins	ISL	2010	345
					26	Unknown	n/a	n/a	n/a
					102	Cummins	ISL	n/a	n/a
					103	Cummins	ISL	2010	380
					104	Cummins	ISL9	2013	345
8a	Urban buses	1	North	6	68	n/a	n/a	n/a	n/a
					69	n/a	n/a	n/a	n/a
					70	n/a	n/a	n/a	n/a
					108	n/a	n/a	n/a	n/a
					109	n/a	n/a	n/a	n/a
					110	n/a	n/a	n/a	n/a
8b	Express buses	1	South	5	93^b^	Cummins	ISL G280	2013	280
					94^b^	Cummins	ISL G280	2013	280
					95^b^	Cummins	ISL G280	2013	280
					96^b^	Cummins	ISL G280	2013	280
					97^b^	Cummins	ISL G280	2013	280
9a	Freeway work	1	Both	5	3	Cummins	ISB6.7 260	2012	260
					4	Cummins	ISB6.7 260	2012	260
					37	Cummins	ISB6.7 260	2012	260
					38	Cummins	ISB6.7 260	2012	260
					62	Cummins	ISB6.7 260	2012	260
9b	Sweeping	1	Both	5	40	Cummins	ISB6.7 280	2012	280
					41	Cummins	ISB6.7 280	2012	280
					42	Cummins	ISB6.7 280	2013	280
					43	Cummins	ISB6.7 280	2012	280
					44	Cummins	ISB6.7 280	2012	280
9c	Municipal work	1	South	3	5	Detroit Diesel	DD13 12.8	2010	500
					6	Cummins	ISB6.7 240	2010	240
					7	Cummins	ISB6.7 240	2010	240
9d	Towing	2	Both	7	45	Cummins	ISX15 550	2012	550
					46	Cummins	ISX15 525	2014	525
					47	Cummins	ISX15 550	2014	550
					48	Paccar	PX-8	n/a	n/a
					105	Cummins	ISB6.7 260	2014	260
					106	Cummins	ISB6.7 280	2013	280
					107	Cummins	ISB6.7 281	2014	280
10	Utility repair	1	North	5	63	Detroit Diesel	DD13	2012	500
					64	Detroit Diesel	DD13	2012	500
					65	Detroit Diesel	DD13	2012	500
					66	Detroit Diesel	DD13	2012	500
					67	Detroit Diesel	DD13	2012	500
	*Total*	*24*		*90*					

a North = Northern California; South = Southern California.

b No SCR temperature data.

All of the 90 vehicles are either commercial class 7 (GVWR 26,001–33,000 lbs) or class 8 (GVWR >33,000 lbs). All the vehicles run on conventional diesel engines except the six urban buses (diesel hybrid electric) and the five express buses (compressed natural gas). Most of the vehicles have engine model year 2010 or newer and are equipped with SCR. There is a good balance between vehicle samples from both regions of California when considering the overall vehicle samples as a whole, although not every vehicle group includes vehicle samples from both regions of the state.

The data were collected using J1939 Mini Logger^TM^, produced by HEM Data, that recorded vehicle speed, position (latitude and longitude), and more than 170 engine and aftertreatment parameters (including engine load and exhaust temperature) at the frequency of one Hz. The data collection effort spanned from November 2014 to September 2016, but was intermittent depending on when the participating fleets were successfully recruited and when the vehicles and data loggers were available. For each vehicle, the data were collected for a minimum period of one month with many vehicles having data collected for several months.
