# Supplementary material for: Real-world exhaust temperature and engine load distributions of on-road heavy-duty diesel vehicles in various vocations
Source: Data Brief. 2018 Apr 18;18:1520–43. doi: 10.1016/j.dib.2018.04.044 (PMC5997961; doi:10.1016/j.dib.2018.04.044)
Supplement: Supplementary file 1 — Supplementary material [file mmc1.pdf]

<u>Author's agreement</u>

We the undersigned declare that the manuscript entitled "**Real-World Exhaust Temperature and Engine Load Distributions of On-Road Heavy-Duty Diesel Vehicles in Various Vocations**" is original, has not been full or partly published before, and is not currently being considered for publication elsewhere.

We confirm that the manuscript has been read and approved by all named authors and that there are no other persons who satisfied the criteria for authorship but are not listed. We further confirm that the order of authors listed in the manuscript has been approved by the undersigned.

We understand that the Corresponding Author is the sole contact for the editorial process. The corresponding author "**Kanok Boriboonsomsin**" is responsible for communicating with the other authors about process, submissions of revisions, and final approval of proofs.

Signature of all authors:

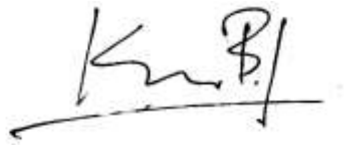

____

Kanok Boriboonsomsin

April 10, 2018
